# Proteomic analysis of HIV-1 Nef cellular binding partners reveals a role for exocyst complex proteins in mediating enhancement of intercellular nanotube formation

**DOI:** 10.1186/1742-4690-9-33

**Published:** 2012-06-22

**Authors:** Joya Mukerji, Kevin C Olivieri, Vikas Misra, Kristin A Agopian, Dana Gabuzda

**Affiliations:** 1 Department of Cancer Immunology and AIDS, Dana Farber Cancer Institute, Boston, MA, USA; 2 Division of Medical Sciences Program in Virology, Harvard Medical School, Boston, MA, USA; 3 Department of Neurology (Microbiology), Harvard Medical School, Boston, MA, USA

**Keywords:** HIV, Nef, Exocyst complex, Intercellular nanotubes, Pak2 kinase, Fluorescence confocal microscopy

## Abstract

**Background:**

HIV-1 Nef protein contributes to pathogenesis via multiple functions that include enhancement of viral replication and infectivity, alteration of intracellular trafficking, and modulation of cellular signaling pathways. Nef stimulates formation of tunneling nanotubes and virological synapses, and is transferred to bystander cells via these intercellular contacts and secreted microvesicles. Nef associates with and activates Pak2, a kinase that regulates T-cell signaling and actin cytoskeleton dynamics, but how Nef promotes nanotube formation is unknown.

**Results:**

To identify Nef binding partners involved in Pak2-association dependent Nef functions, we employed tandem mass spectrometry analysis of Nef immunocomplexes from Jurkat cells expressing wild-type Nef or Nef mutants defective for the ability to associate with Pak2 (F85L, F89H, H191F and A72P, A75P in NL4-3). We report that wild-type, but not mutant Nef, was associated with 5 components of the exocyst complex (EXOC1, EXOC2, EXOC3, EXOC4, and EXOC6), an octameric complex that tethers vesicles at the plasma membrane, regulates polarized exocytosis, and recruits membranes and proteins required for nanotube formation. Additionally, Pak2 kinase was associated exclusively with wild-type Nef. Association of EXOC1, EXOC2, EXOC3, and EXOC4 with wild-type, but not mutant Nef, was verified by co-immunoprecipitation assays in Jurkat cells. Furthermore, shRNA-mediated depletion of EXOC2 in Jurkat cells abrogated Nef-mediated enhancement of nanotube formation. Using bioinformatic tools, we visualized protein interaction networks that reveal functional linkages between Nef, the exocyst complex, and the cellular endocytic and exocytic trafficking machinery.

**Conclusions:**

Exocyst complex proteins are likely a key effector of Nef-mediated enhancement of nanotube formation, and possibly microvesicle secretion. Linkages revealed between Nef and the exocyst complex suggest a new paradigm of exocyst involvement in polarized targeting for intercellular transfer of viral proteins and viruses.

## Background

The Nef protein of human and simian immunodeficiency viruses plays an important role in disease pathogenesis and progression to AIDS [1-5]. HIV-1 Nef is a 27 kDa phosphoprotein that is membrane-associated via N-terminal myristoylation. Diverse functions of Nef include downregulation of CD4 and MHC-I [4], enhancement of viral replication and infectivity [1], modulation of T-cell signaling [6-8], proliferation of multivesicular bodies (MVBs) [4,9-11], and induction of nanotube formation [12,13].

Nef has been linked to intracellular trafficking via interactions with the endocytic and exocytic host cell machinery [4]. Nef mediates downregulation of CD4 and MHC-I via well-characterized mechanisms. Nef downregulates CD4 by bridging between CD4 and the AP-1/AP-2 and/or AP-3 adapter proteins of clathrin-coated pits, accelerating CD4 endocytosis; Nef also redirects endosomes to MVBs prior to lysosomal degradation of CD4 [14-18]. CD4 downregulation enhances viral replication by preventing superinfection and reducing interference with envelope glycoprotein functions. Nef disrupts constitutive recycling of MHC-I by interacting with PACS-1 and diverting MHC-I from recycling endosomes to the trans Golgi network [19,20], which allows infected cells to evade cytotoxic T-cell surveillance. Fusion of MVBs with the plasma membrane liberates vesicles, known as exosomes, into the extracellular space [21]. Secreted forms of Nef are associated with exosomes and/or exosome-like microvesicles, which can enhance activation or apoptosis of bystander cells, suggesting a potential role in pathogenesis [22-27].

Nef mediates enhancement of T-cell activation via unknown mechanisms that involve activation of Pak2 (p21-activated kinase 2) in a multiprotein Nef-Pak2 complex [28-33]. Components of the ~1 MDa Nef-Pak2 multiprotein complex include Nef, Pak2, Vav, Cdc42, possibly β-PIX, PI-3-kinase, and other unknown proteins [31,34-37]. The Src-family kinases (SFKs) Lck and Fyn play a proximal role in T-cell activation, as they are the first kinases recruited to the TCR upon antigen binding [1]. Nef has been shown to interact with the SH3 domains of these SFKs, potentially implicating them in Nef-mediated enhancement of T-cell activation [1,38,39]. Pak2 activation is required for IL-2 secretion and NFAT signaling in activated T-cells, and Nef-mediated enhancement of cellular activation and viral replication in primary T-cells [33,40]. Pak2 regulates actin cytoskeleton dynamics to mediate several functions, including localization of T-cell signaling microclusters (comprised of TCR-CD3 complexes, ZAP-70 and SFKs, and adaptors such as LAT), which in turn influences formation of the immunological synapse [41-43]. Because activated T-cells upregulate exosome secretion [44], it is unclear whether enhanced secretion of exosomes in the presence of Nef is a direct result of Nef’s association with these microvesicles or an indirect consequence of Nef-mediated enhancement of T-cell activation.

Nef influences immunological synapse formation; however, studies reached different conclusions regarding whether or not Nef inhibits this process. Studies in Jurkat cells indicated that HIV-1 Nef impairs formation of immunological synapses by inhibiting recycling of endocytosed TCR-CD3 complexes and Src family kinases to the plasma membrane and via mislocalization of N-Wasp [41,45,46]. However, inhibitory effects of HIV-1 Nef on immunological synapse formation were not observed in other studies [6,47]. While Nefs from most non-human primate immunodeficiency viruses inhibit immunological synapse formation by downregulating CD28 and TCR-CD3 complexes, SIVcpz and HIV-1 Nefs do not exhibit this effect [47]. Furthermore, without altering the quantity or assembly rate of immunological synapses, HIV-1 Nef expression more than doubled the number of Jurkat E6-1 cells activated by TCR stimulation, possibly due to rapid recruitment of Nef in lipid rafts to the immunological synapse [6]. Possible explanations for divergent conclusions regarding effects of HIV-1 Nef on immunological synapse formation include differences in Nef expression levels, the time point at which immunological synapse formation was analyzed, and Nef alleles or cell-type studied.

In addition to its effects on formation of immunological synapses, Nef has been shown to stimulate formation of other types of intercellular contacts, including virological synapses and tunneling nanotubes [12,42]. Actin- and microtubule-dependent clustering of adhesion molecules and membrane microdomains at virological synapses leads to formation of short-range tubes through which Gag, Env, and HIV-1 can traffic between cells by hijacking cellular transport mechanisms [42]. Nef is required for efficient HIV replication in co-cultures of dendritic cells and T-cells [48], which is mediated by virological synapses [42] and other modes of intercellular transfer [49]. In contrast to virological synapses, tunneling nanotubes are longer-range narrow intercellular conduits that form independent of receptor contacts [42,50]. HIV-1 transfer between CD4+ T-cells and Nef transfer from infected macrophages to B-cells via nanotubes have been reported [13,51]. Nanotube formation between macrophages and B-cells is Nef-dependent, and has been proposed to suppress NF-κB-induced class-switch recombination by transferring Nef to B-cells [10,13].

Polarized transfer of Nef between multiple cell-types of the immune system suggests that Nef exploits host cell trafficking machinery to maximize its spread. Here, we report that Nef associates with 5 of the 8 components of the exocyst complex, an octameric protein complex that targets vesicles to the plasma membrane, regulates polarized exocytosis, and recruits membranes and proteins required for nanotube formation. Furthermore, shRNA-mediated depletion of EXOC2, an exocyst complex subunit, abolishes Nef-mediated enhancement of nanotube formation. Association of Nef with the exocyst complex likely plays a role in several proposed functions of Nef, including Nef-mediated enhancement of microvesicle secretion and nanotube formation, thereby contributing to cell-cell spread of viral infection and chronic immune activation during AIDS pathogenesis.

## Results

### Identification of cellular proteins associated with wild-type Nef but not mutant Nefs defective for Pak2 association

To better understand interactions of Nef with the cellular trafficking machinery, we performed mass spectrometry analysis of Nef-associated proteins using wild-type Nef and Nef mutants defective for association with the Pak2-activating complex. Pak2 regulates cytoskeletal rearrangements and interacts with several proteins that control cell motility, membrane ruffles, and filipodia, including Rac1, Cdc42, and β-PIX [52]. Therefore, we used the wild-type 5C Nef, a primary Nef previously characterized as a strong activator of Pak2, and two well-characterized 5C-mutants that have wild-type abilities to downregulate CD4 but are defective for the ability to associate with and activate Pak2: 5C-7 (F85L, F89H, H191F in NL4-3) and 5C-AxxA (A72P, A75P) [53]. 5C-7 Nef is fully functional for MHC-I downregulation [53], while 5C-AxxA is defective for this function. Disrupting the SH3-binding PxxP motif at Nef residues 72 to 75 also abrogates SH3-dependent interactions of Nef with SFKs and Vav. Thus, 5C-7 and 5C-AxxA allow investigation of Nef-dependent effects on protein trafficking mediated via Pak2 activation and/or SH3 binding.

**Figure 1:**
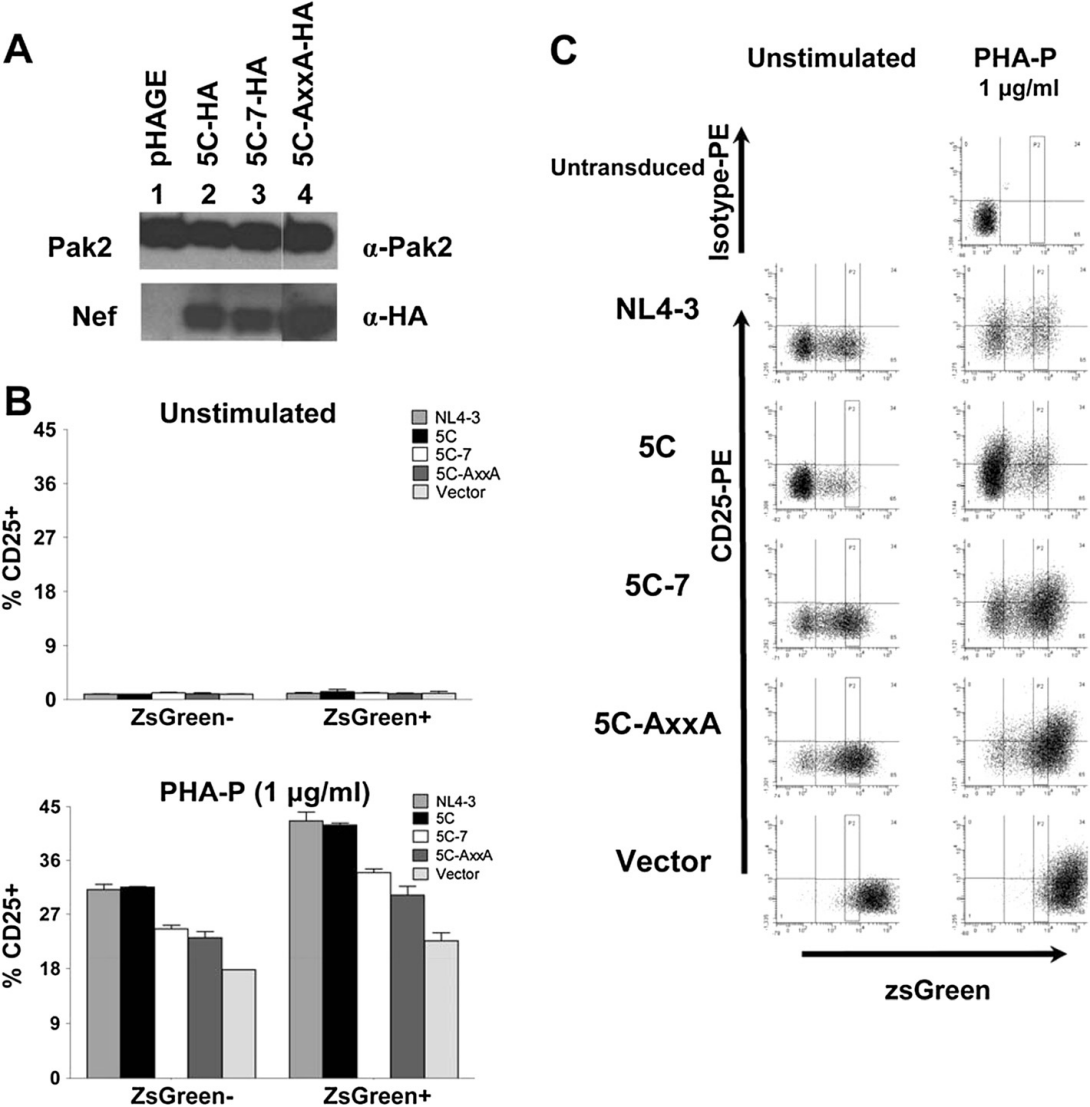
** Nef mutants defective for association with Pak2 are also defective for the ability to enhance T-cell activation upon PHA stimulation.A)** Jurkat E6-1 cell lysates were harvested at 48 hpi following transduction with VSV-G pseudotyped pHAGE-Nef-HA-IRES-ZsGreen vector virions encoding wild-type (Lane 2) or mutant Nefs (Lanes 3 and 4), or empty vector (Lane 1), separated by SDS-MOPS-PAGE, and immunoblotted to detect HA-tagged Nef and endogenous Pak2. Lane 4 is a non-contiguous lane from the same gel as Lanes 1–3. For clarity, brightness and contrast of the Nef panels were increased by 2%. **B**) CD25 expression on Jurkat E6-1 cells transduced with VSV-G pseudotyped pHAGE-EF1α-IRES-ZsGreen vector encoding wild-type (NL4-3 or 5C) or mutant (5C-7, 5C-AxxA) Nef or empty vector was analyzed at 48 h post-transduction, with (lower panel) or without (upper panel) 1 μg/mL PHA-P stimulation for 24 h prior to FACS analysis. ZsGreen expression is a reporter for pHAGE transduction and Nef expression, except in the case of vector samples, which lack Nef. For each sample, the Nef allele encoded in the pHAGE-IRES-ZsGreen vector virions is indicated (see legend). Bars graphed on the left and right of each panel represent the ZsGreen-negative and ZsGreen-positive subsets of cells from each transduction. **C**) FACS plots and gating strategy for panel B are shown. The gate “P2” demarcates populations of matched ZsGreen fluorescence for which percentages of CD25-positive cells are reported in panel B.

First, we constructed pHAGE lentiviral vectors encoding Nef-IRES-ZsGreen under the control of an EF1-α promoter, to express Nef in the absence of other viral proteins and allow identification and sorting of Nef-expressing cells. Western blotting confirmed that Jurkat E6-1 cells transduced with these vectors express similar levels of HA-tagged wild-type or mutant Nefs after gating on cell populations with matched mean ZsGreen fluorescence and FACS sorting (Figure 1A). Relative to the wild-type and reference Nefs 5C and NL4-3, which increased the percentage of CD25-expressing cells by ~2-fold, the 5C-7 and 5C-AxxA Nef mutants exhibited a modest but reproducible reduction in Nef-mediated enhancement of Jurkat cell activation, as indicated by the percentage of pHAGE-transduced cells that upregulate CD25 following treatment with a sub-threshold stimulus for T-cell activation (1 μg/mL PHA-P; Figure 1B lower panel and Figure 1C right column). Due to a bystander effect (i.e., paracrine activation of ZsGreen-negative Nef-minus cells, possibly mediated by IL-2 and/or Nef secreted from co-cultured Nef-IRES-ZsGreen-expressing cells), some CD25 upregulation was also observed in the Nef-minus (ZsGreen-negative) samples (Figure 1B, left). The lower levels of CD25 expression in cells bearing 5C-7 and 5C-AxxA Nefs are consistent with the requirement for Pak2 in Nef-mediated enhancement of T-cell activation [33], and decreased ability of these mutant Nefs to activate Pak2 demonstrated by Agopian *et al*. [53]. Although the ~2-fold increase in the percentage of cells expressing CD25 (Figure 1B, vector-transduced Nef- vs. 5C-transduced Nef+) is a modest effect, these results are consistent with prior studies of Nef-mediated enhancement of Jurkat cell activation [7,33,54] and may reflect high background activation in these immortalized cells. We also tested additional T-cell activation markers, CD69 and HLA-DR, in this assay system. However, CD69 expression was similarly high (98.7 ± 1.0% with Nef versus 95.2 ± 0.6% without Nef, SEM, data not shown), and HLA-DR expression remained low irrespective of Nef expression in pHAGE-transduced Jurkat cells (8.8 ± 0.1% versus 7.1 ± 0.1%, SEM, data not shown). Therefore, CD25 was used as the readout for Nef-mediated enhancement of T-cell activation based on the increased percentage of CD25-positive cells and increase in mean fluorescence intensity (MFI) (Figure 1B and C).

**Figure 2:**
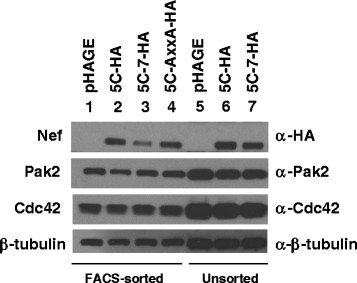
**Nef-Pak2 complex proteins are expressed at comparable levels in pHAGE-Nef transduced Jurkat cells utilized for mass spectrometry.** Proteins immunoblotted in 1% of the input lysates utilized for LC-MS/MS analysis are shown. Jurkat E6-1 cell lysates were harvested 48 h after transduction with VSV-G pseudotyped pHAGE-Nef-HA-IRES-ZsGreen vector virions encoding wild-type wild-type (Lane 2 and 6) or mutant Nefs (Lanes 3, 4, and 7), or empty vector (Lanes 1 and 5). Lysates analyzed in Lanes 5–7 were derived from unsorted cells, while lysates in Lanes 1–4 were from cells sorted to obtain populations with matched ZsGreen fluorescence. β-tubulin is included as a loading control.

To identify cellular proteins that associate with wild-type Nef but not mutant Nefs defective for Pak2 association, we performed tandem mass spectrometry analysis of Nef-associated proteins following immunoprecipitation of Nef from unsorted cells (53–67% ZsGreen-positive) transduced with pHAGE-EF1α-IRES-ZsGreen encoding HA-tagged 5C, 5C-7, or 5C-AxxA, or from sorted populations of Jurkat cells (100% ZsGreen-positive) isolated via high-speed FACS after gating on cells with matched mean ZsGreen fluorescence. Immunoblotting an aliquot of the input lysate utilized for co-immunoprecipitation and mass spectrometry indicated that components of the Nef-Pak2 multiprotein complex were expressed at comparable levels in the sorted and unsorted samples (Figure 2, Lanes 1–4 and 5–7). Jurkat cells expressing the empty vector pHAGE-EF1α-IRES-ZsGreen were included as a negative control. Nef immunocomplexes were eluted with HA peptide, trichloroacetic acid-precipitated, trypsinized, purified, and analyzed via LC-MS/MS. Mass spectrometry analysis identified peptides from ~65 to 135 proteins in each of the 7 samples (Additional file 1: Table S1). Approximately 50 of these proteins were detected in all samples, including empty vector and mutant Nef samples (Additional file 1: Table S1 and Additional file 2: Table S2). Consistent with their prior detection as Nef-interacting proteins [29], peptides mapping to DOCK2 and ELMO1 were identified at high abundance (57 to 91 and 9 to 17 peptides per sample, respectively), and received high scores from CompPASS (Comparative Proteomics Software Suite [55]) analysis (Z = 10.1 for each, and D^N^ = 33.72 and 9.21 for DOCK2 and ELMO, respectively) (Additional file 3: Figure S1, Additional file 1: Table S1 and Additional file 2: Table S2). The normalized D-score, D^N^, provides a measure of the uniqueness and reproducibility of an interaction, and the abundance of peptides detected from a given interactor [56]. However, DOCK2 and ELMO1 were detected in all samples, irrespective of whether or not the sample contained Nef, and thus could not be considered Nef-interacting proteins. Filtering the mass spectrometry results to omit proteins that were detected in mutant and empty-vector samples yielded 10 proteins specifically associated with wild-type Nef (Table 1).

**Table 1:** Cellular proteins detected exclusively in wild-type Nef immunocomplexes

Accession	Gene	Protein	Description		Unique # peptides
*FACS-sorted 5C Nef*					
Q9NV70	*EXOC1*	EXOC1	Exocyst complex component 1, isoform 1		3
Q96KP1	*EXOC2*	EXOC2	Exocyst complex component 2		3
O60645	*EXOC3*	EXOC3	Exocyst complex component 3, isoform 1		3
Q96A65	*EXOC4*	EXOC4	Exocyst complex component 4		6
Q8TAG9	*EXOC6*	EXOC6	Exocyst complex component 6		1
Q9UL85 ^a^			Myosin-reactive Igκ chain variable region (fragment)		1
P19838	*NFKB1*	NFKB1	Nuclear factor NF-κB p105 subunit, isoform 2		1
*Unsorted 5C Nef*					
Q9NV70	*EXOC1*	EXOC1	Exocyst complex component 1, isoform 1		3
Q96KP1	*EXOC2*	EXOC2	Exocyst complex component 2		3
O60645	*EXOC3*	EXOC3	Exocyst complex component 3, isoform 1		2
Q96A65	*EXOC4*	EXOC4	Exocyst complex component 4		4
Q9NP29 ^a^			Microfibrillar protein 2 (fragment)		1
Q13177	*PAK2*	Pak2	PAK2 Serine/threonine-protein kinase		3
P60866	*RPS20*	RS20	40S ribosomal protein S20		1
***Gene or Accession***	**D**^**N**^**-score**^b^	**Z-score**^b^	**Description**	**Total #peptides**	**Unique # peptides**
*FACS-sorted and unsorted 5C Nef combined*					
*EXOC1*	7.14	10.09	Exocyst complex component 1, isoform 1	6	5
*EXOC2*	7.14	10.09	Exocyst complex component 2	6	5
*EXOC3*	5.83	10.09	Exocyst complex component 3, isoform 1	4	4
*EXOC4*	9.21	10.10	Exocyst complex component 4	10	6
*EXOC6*	0.41	10.05	Exocyst complex component 6	1	1
*NFKB1*	0.05	−0.59	Nuclear factor NF-κB p105 subunit, isoform 2	1	1
*PAK2*	0.70	10.09	PAK2 Serine/threonine-protein kinase	3	3
Q9NP29 ^a^	0.41	10.05	Microfibrillar protein 2 (fragment)	1	1
Q9UL85 ^a^	0.41	10.05	Myosin-reactive Igκ chain variable region (fragment)	1	1
*RPS20*	0.04	−1.51	40S ribosomal protein S20	1	1

a) Unreviewed entries from UniProtKB/TrEMBL; all other accessions from Swiss-Prot/UniProtKB [57]. b) Statistical scores obtained via CompPASS analysis.

### Nef associates with exocyst complex proteins

Proteins detected via LC-MS/MS analysis of wild-type 5C Nef-HA immunocomplexes but absent from background or mutant control samples are shown in Table 1; upper and lower panels describe individual samples and combined data, respectively. Pak2 was identified in wild-type Nef, but not mutant Nef or empty vector samples (Table 1 and Additional file 1: Table S1 and Additional file 2: Table S2), consistent with prior studies that identified Pak2 as a Nef-associated protein and the 5C-7 and 5C-AxxA mutants as being defective for association with Pak2 [53]. EXOC1, EXOC2, EXOC3, and EXOC4, 4 of the 8 proteins that comprise the exocyst complex [58,59], were identified in 2 independent wild-type Nef immunoprecipitation samples, and in none of the 5 control samples (Table 1 and Additional file 1: Table S1 and Additional file 2: Table S2). In addition, EXOC6 was detected in the sorted 5C Nef sample (Table 1). CompPASS comparative proteomic analysis software suite [55] indicated that Nef-association of EXOC1, EXOC2, EXOC3, EXOC4, EXOC6 and Pak2 is statistically significant according to Z-scores and/or normalized D-scores (D^N^) (Table 1, Additional file 3: Figure S1, Additional file 1: Table S1 and Additional file 2: Table S2). Variation in whether individual proteins were detected solely in sorted or unsorted cells may reflect stochastic fluctuation in peptides contacting the detector during mass spectrometric analysis. Therefore, detection of EXOC1, EXOC2, EXOC3, and EXOC4 in two independent mass spectrometry samples (i.e., immunocomplexes from sorted and unsorted cells) raises confidence in Nef-exocyst complex association. Thus, 5 proteins of the exocyst complex were associated with wild-type Nef, but not mutant Nefs or empty vector negative controls.

In addition to Pak2 kinase and the 5 exocyst complex components, 4 other proteins were detected exclusively in immunocomplexes containing wild-type Nef: p105 subunit of NF-κB, variable region of myosin-reactive Igκ chain, microfibrilliar protein 2, and 40S ribosomal subunit (Table 1). Some studies suggest involvement of Nef in HIV-mediated enhancement of NF-κB induction [60], while others did not observe NF-κB activation when Nef was expressed in the absence of other HIV proteins [61,62]. Therefore, detection of NF-κB in 5C Nef immunocomplexes from sorted cells may reflect cytoplasmic retention of NF-κB [13], rather than specific association of NF-κB with Nef. The 40S ribosomal subunit was likely detected due to its high abundance in cells; however, exocyst-mediated effects on protein synthesis and translation have been reported [63]. Peptides matched to the variable region of myosin-reactive Igκ chain and microfibrilliar protein 2 were identified with lower confidence, as these were single-peptide hits corresponding to protein fragments in a subdivision of UniProt that is not reviewed (UniProt/TrEMBL), as compared to UniProt/SwissProt, which is manually curated [57]. The finding that Pak2 was identified only in unsorted cells, while NF-κB was detectable only in sorted cells, may reflect stochastic fluctuation in peptides contacting the detector.

**Figure 3:**
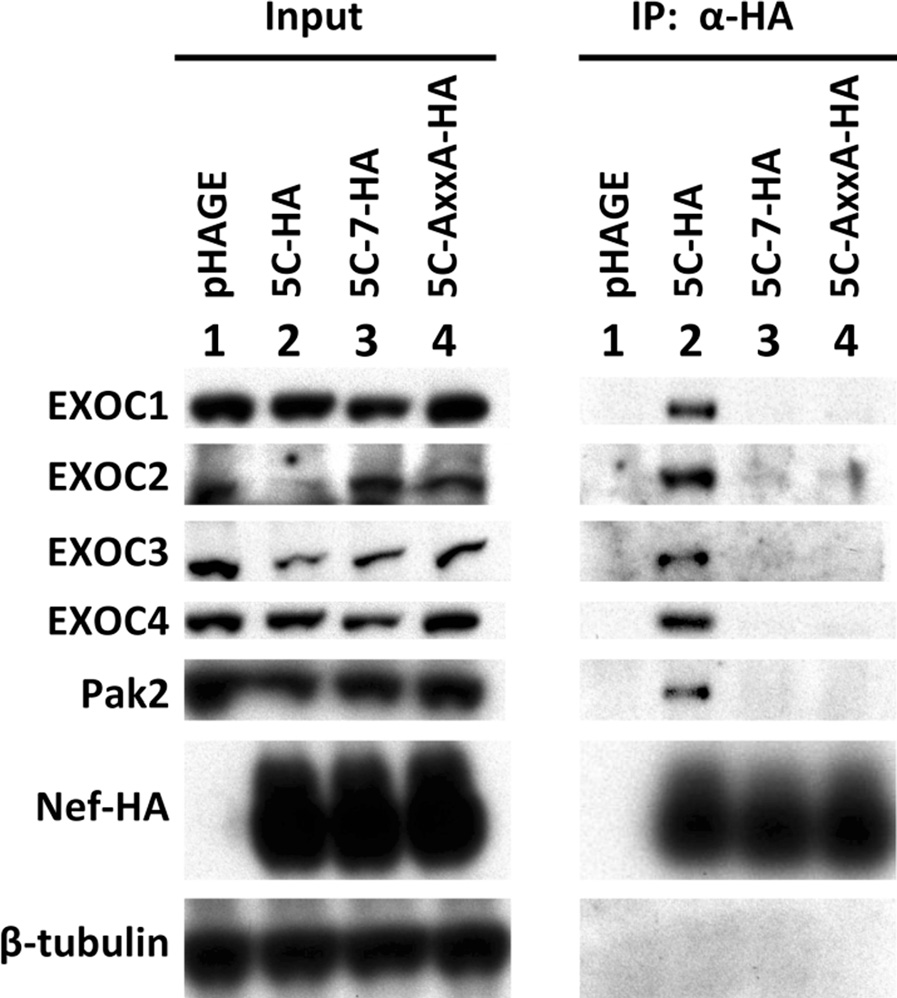
**Exocyst complex components EXOC1, EXOC2, EXOC3, and EXOC4 co-immunoprecipitate with wild-type, but not mutant Nefs, in Jurkat E6-1 cell lysates.** Nef-HA immunocomplexes (right) and input lysates (left) from Jurkat E6-1 cells harvested at 48 h post-transduction with pHAGE virions bearing wild-type (5C, Lane 2) or mutant Nefs (5C-7, 5C-AxxA; Lanes 3 and 4) or empty pHAGE-IRES ZsGreen vector (Lane 1) were immunoblotted to detect endogenous EXOC1, EXOC2, EXOC3, EXOC4, Pak2, and HA-tagged Nef. β-tubulin is included as a loading control.

To verify the association of Nef with EXOC1, EXOC2, EXOC3, and EXOC4 revealed by mass spectrometry analysis, we performed a co-immunoprecipitation assay with 5C, 5C-7, and 5C-AxxA Nefs from pHAGE-transduced Jurkat cells and analyzed the co-immunoprecipitated proteins via Western blotting (Figure 3). The results confirmed that endogenous EXOC1-4 associate with 5C Nef in Jurkat cell lysates (Figure 3, Lane 2). In contrast, association with the mutant Nefs 5C-7 and 5C-AxxA was undetectable for EXOC1, EXOC3, and EXOC4, and barely detectable for EXOC2, compared to the control sample lacking Nef (Figure 3, Lanes 3 and 4 versus Lane 1). Pak2 and β-tubulin were immunoblotted as positive and negative controls for Nef co-immunoprecipitation, respectively. These co-immunoprecipitation results provide validation for the mass spectrometry analysis of Nef-associated proteins, confirming that Nef associates with EXOC1-4.

### Network analysis of Nef-exocyst interactome identifies functional linkages

**Figure 4:**
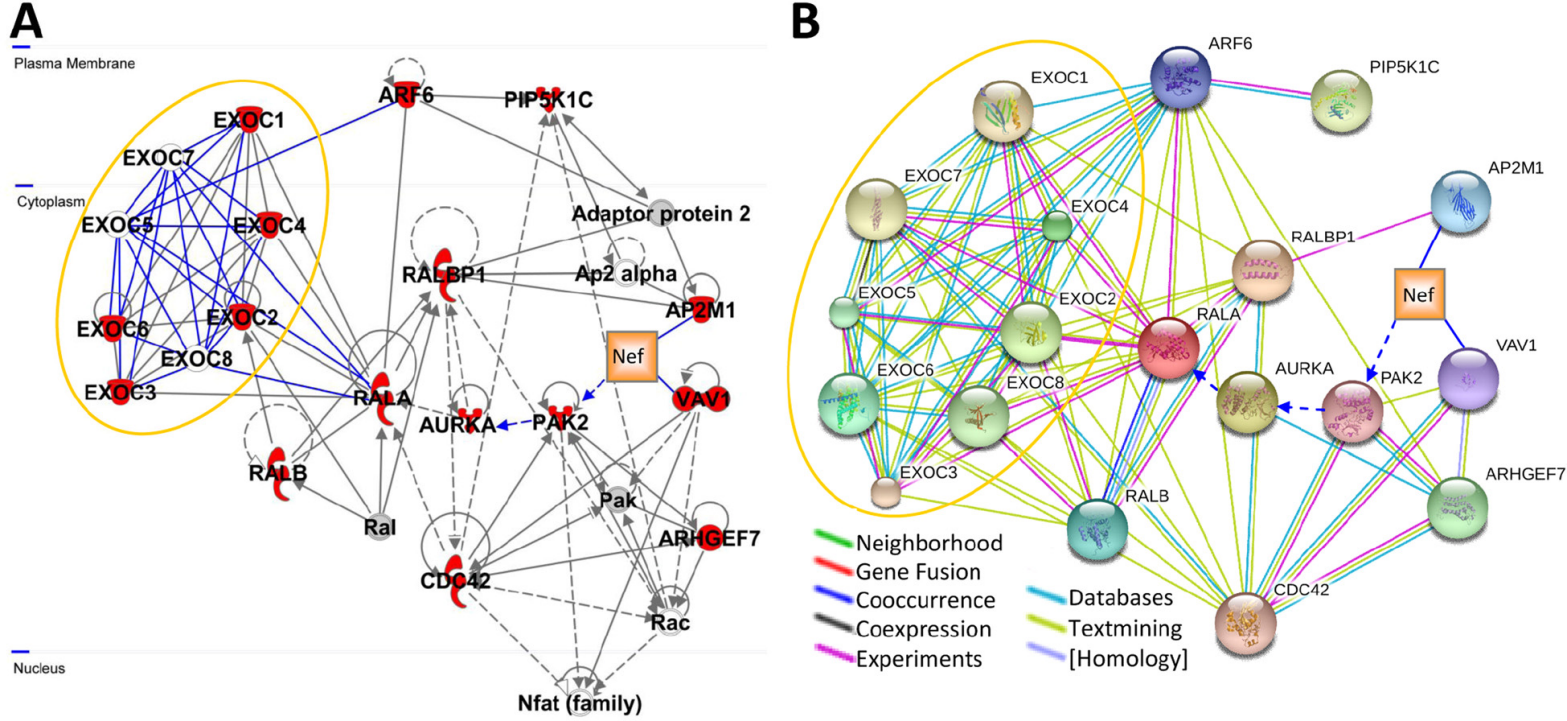
** Visualization of protein interaction networks of Nef-interacting proteins and exocyst complex components, regulators, and effectors.A**) IPA network of Nef-interacting proteins and exocyst complex components, regulators, and effectors. Inputting 16 molecules (depicted as red nodes, described in the text) into Ingenuity Pathways Analysis [Ingenuity® Systems, www.ingenuity.com] yielded a network of 35 molecules, of which 13 [Calmodulin, Dock1-Pak, ERK1/2, Exocyst, Jnk, NFkB (complex), Phosphatidylinositol 4,5 kinase, PI3K (complex), PIP4K2C, Pkc(s) Pld, Ras, and TCR] were omitted for clarity. Components of the exocyst complex are encircled by a gold oval. Manually-curated edges are shown in dark blue, and dashed arrows denote activation via indirect association. IPA assigned this network a score of 42, which is based on the hypergeometric distribution and is the -log(right-tailed Fisher’s exact test result). The score implies a 1 in 10^42^ chance of obtaining a network containing at least the same number of network eligible molecules by chance when randomly picking 16 molecules that are present in networks from the Ingenuity Knowledge Base. **B**) STRING network of Nef-interacting proteins and exocyst complex components, regulators, and effectors. Inputting 19 molecules (16 molecules input into IPA, plus EXOC5, EXOC7, and EXOC8; depicted as spheres) into STRING yielded a network visualizing linkages between Nef, the exocyst complex, and regulators of the actin cytoskeleton. Components of the exocyst complex are encircled by a gold oval. Manually-curated edges are shown in dark blue, and dashed arrows denote activation via indirect association. Other edge colors reflect the evidence channels (see legend) by which protein nodes are related in the STRING database. Scores STRING assigned to relationships depicted in this network are provided in Additional file 4: Table S3.

To visualize functional interactions between Nef and the exocyst complex, we generated protein-association networks using bioinformatic tools. Three well-characterized Nef interactors (Pak2, Vav, and AP2M1, the μ-subunit of adapter protein 2), regulators and effectors of the exocyst complex (RalA, Ral-binding protein, Aurora-A, phosphatidylinositol-4-phosphate 5-kinase type I γ, Cdc42, PIX, and Arf6), and exocyst complex components detected via mass spectrometry (EXOC1, EXOC2, EXOC3, EXOC4, and EXOC6) were selected as input molecules to generate a network (Figure 4A) using Ingenuity Pathway Analysis (IPA) [Ingenuity® Systems]. IPA predicted that all of the input molecules are connected in a single, high-scoring network that links cell signaling, lipid metabolism, and endosomal trafficking, in particular endocytosis and endocytic recycling (Figure 4A). RalA, the adapter protein 2 complex, Pak2, and Vav/Cdc42 represent major hubs of functional interaction between Nef and the exocyst complex (gold oval, Figure 4A). To visualize interactions by which the Nef-exocyst interactome interfaces with regulators of Pak2 and the cytoskeleton, we created an additional network (Figure 4B) using STRING, a bioinformatic tool that maps protein-protein associations based on evidence channels (see Figure 4B key) including comparative genomics, experimental data, pathway databases, natural language processing (e.g., textmining for co-occurrence of protein names in PubMed abstracts), and homology [64]. The STRING network (Figure 4B and Additional file 4: Table S3) visualized protein-protein association relationships that link Nef and the exocyst complex to regulators of endocytosis, cytoskeletal remodelling, lipid metabolism, and membrane trafficking, and identified Cdc42, RalA, and Arf6 as key nodes of functional interaction between Nef and the exocyst complex. These findings are consistent with the network predicted by IPA (Figure 4A), prior reports suggesting that Nef-mediated regulation of Arf6 plays a role in modulating clathrin-dependent endocytosis [3,19], and known interactions of Arf6 with the exocyst complex that mediate membrane recruitment to polarized plasma membrane sites [65].

### EXOC2 depletion abrogates Nef-mediated enhancement of nanotube formation

**Figure 5:**
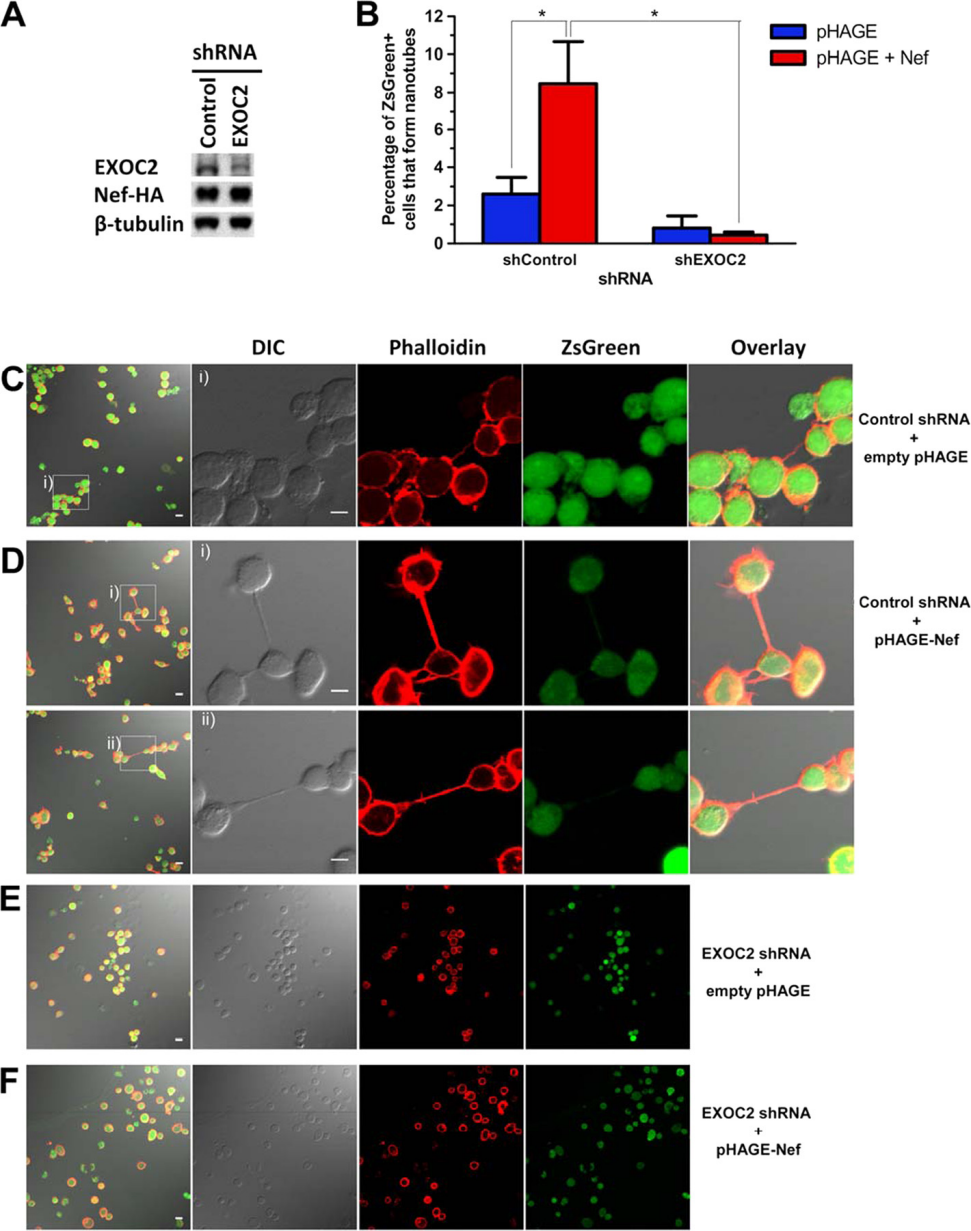
**EXOC2 depletion abrogates Nef-mediated enhancement of nanotube formation.A)** Lysates of Jurkat E6-1 cells transduced with vector virions bearing pLKO.1-shRNA followed by pHAGE-Nef-IRES-ZsGreen encoding 5C (wild-type) or empty pHAGE were harvested 32 h post-pHAGE transduction and immunoblotted to detect endogenous EXOC2, HA-tagged Nef, and β-tubulin. Samples were treated with an shRNA targeting EXOC2 (right lane, EXOC2) or a control shRNA that does not target the human transcriptome (left lane, Control). **B -F)** Jurkat cells transduced as described above were stimulated with 1 ug/mL PHA-P for 1 h at 24 h post-pHAGE transduction, incubated on fibronectin-coated coverslips for 5 h, fixed at 30 h post-pHAGE transduction and phalloidin-stained prior to visualization by confocal fluorescence microscopy. **B**) Percentages of ZsGreen-positive Jurkat cells that formed nanotubes and nanotube-like structures in populations treated with shEXOC2 (right) or shControl (left) are shown. Differences betweenNef-expressing samples treated with control versus EXOC2 shRNAs were significant, as were differences between control shRNA-treated cells with versus without Nef expression (“*” denotes p < 0.05, Mann–Whitney U). The percentage of Nef-expressing cells that formed nanotubes and nanotube-like structures was calculated by manually counting the number of connected cells and dividing the result by the total number of ZsGreen-positive cells for a given sample. The total numbers of ZsGreen-positive cells counted in 30 fields at 40X magnification are detailed in the Methods. Data represent percentages of ZsGreen-positive cells connected by nanotubes, and are reported as mean ± SEM. **C**-**F**) Selected fields that contain nanotubes (**C** and **D**) and representative confocal fluorescence images (**E** and **F**) from the samples quantified in B are shown. Actin-containing nanotubes and nanotube-like structures were visualized via Alexa Fluor 555-conjugated phalloidin; pHAGE-transduced cells were detected via ZsGreen fluorescence. Scale bars, 5 μm.

The exocyst complex mediates intercellular nanotube formation [66]. Given that Nef has been reported to enhance nanotube formation [13], we sought to determine whether the exocyst complex is an effector in Nef-mediated enhancement of nanotube formation. To address this question, we performed pLKO.1-shRNA knockdowns targeting EXOC2 in Jurkat cells, transduced these cells with pHAGE-Nef-IRES-ZsGreen or empty vector, and assayed for nanotube formation using confocal fluorescence microscopy-based detection of actin and the Nef reporter ZsGreen (Figure 5). Treatment of Jurkat cells with an shRNA targeting EXOC2 decreased EXOC2 expression by at least 2-fold, compared to treatment with a control shRNA that does not target any human transcripts (Figure 5A, “EXOC2” versus “Control”). We observed percentages of nanotube-forming cells comparable to those reported by others for Jurkat E6-1 cells [51,67]. In pHAGE-transduced populations expressing comparable levels of Nef (Figure 5A), greater than 8% of ZsGreen-positive, Nef-expressing cells formed nanotubes and nanotube-like structures after treatment with a control shRNA, whereas the corresponding value for EXOC2 knockdown cells was < 0.4% (Figure 5B). The control shRNA cells expressing Nef exhibited morphological changes consistent with cytoskeletal rearrangements and cell polarization (Figure 5D): these cells formed at least 3-fold more nanotubes and nanotube-like structures compared to corresponding cells lacking Nef. Moreover, in Nef-expressing cells, nanotubes were more robust and occurred more frequently, in some cases with multiple connections originating from a single cell (approximately 5% of nanotube-forming cells) (Figure 5B and 5D, and Additional file 5: Figure S2). In contrast, Jurkat cells expressing an shRNA targeting EXOC2 formed few, if any, nanotubes, irrespective of whether or not Nef was expressed (Figure 5E and 5F). Thus, depletion of EXOC2 abrogated Nef-mediated enhancement of nanotube formation (Figure 5B; p < .05, Mann–Whitney U). These findings suggest that exocyst complex function is required for Nef-mediated enhancement of nanotube formation.

## Discussion

Our findings demonstrate a novel association of Nef with the host cell exocytic machinery that has implications for understanding mechanisms involved in intercellular transfer of Nef and other HIV-1 proteins. We identified EXOC1, EXOC2, EXOC3, EXOC4, and EXOC6 as Nef-associated proteins via mass spectrometry analysis of Nef immunocomplexes isolated from Jurkat cells, and showed this association was disrupted by mutations that abrogate the ability of Nef to associate with and activate Pak2 kinase. Furthermore, association of wild-type, but not mutant Nef, with EXOC1, EXOC2, EXOC3, and EXOC4 was verified by co-immunopreciptation assays in Jurkat cells. Importantly, shRNA-mediated depletion of EXOC2 abrogated Nef-mediated enhancement of nanotube formation in Jurkat cells. Together, these results suggest that the exocyst complex is likely to be a key effector mediating Nef’s ability to promote nanotube formation, and may mediate some of its other functions as well (e.g. microvesicle secretion). DOCK2 and ELMO1 were previously reported as Nef-interacting proteins [29]; however, we detected DOCK2 and ELMO1 in all of the mass spectrometry samples, including empty vector control samples, and the number of peptides detected did not differ significantly in the presence or absence of Nef, suggesting these proteins are not specifically associated with Nef (Additional file 1: Table S1 and Additional file 2: Table S2).

Our finding that Nef associates with EXOC1-4 and EXOC6 in Jurkat cells is consistent with a recent publication from the Krogan lab, which reported that Nef associates with EXOC4 based on affinity tagging and mass spectrometry analysis using a different Nef allele (HxB2 Nef fused to a 2X Strep and 3X FLAG purification tag) expressed in Jurkat cells [17]. In this study, MiST (mass spectrometry interaction statistics) assigned this interaction a score of .769, where a threshold of .75 indicates significance [17]. The authors were not able to reproduce Nef-EXOC4 association by co-immunoprecipitation assays in transfected 293T cells, however, raising the possibility that a cell-type-specific factor(s) may be required for Nef-EXOC4 association [17].

Nef-mediated enhancement of T-cell activation requires stimulation via TCR or PMA/PHA (Figure 1B and 1C). Our proteomic analysis was performed using unstimulated Jurkat cells. Moreover, FACS analysis of CD25 expression detected little or no expression of T-cell activation markers (Figure 1B upper panel and 1C). These observations suggest that the enhanced nanotube formation and secretion of microvesicles from Nef-expressing cells reported in prior studies [13,22-24] may be due to a direct effect of Nef on the exocytic machinery, rather than an indirect effect of Nef-mediated enhancement of T-cell activation. Microarray data from Jurkat cells expressing SIV Nef indicated that exocyst complex components were upregulated only 1.5 to 2-fold [68], and gene expression profiles of CD4+ T-cells from HIV-infected patients compared to uninfected individuals showed only minor effects on levels of transcripts encoding exocyst complex proteins [69]. Thus, our mass spectrometry data is likely to reflect direct or indirect association of Nef with the exocyst complex, rather than Nef-induced enhancement at the level of gene expression or enhancement of T-cell activation.

**Figure 6:**
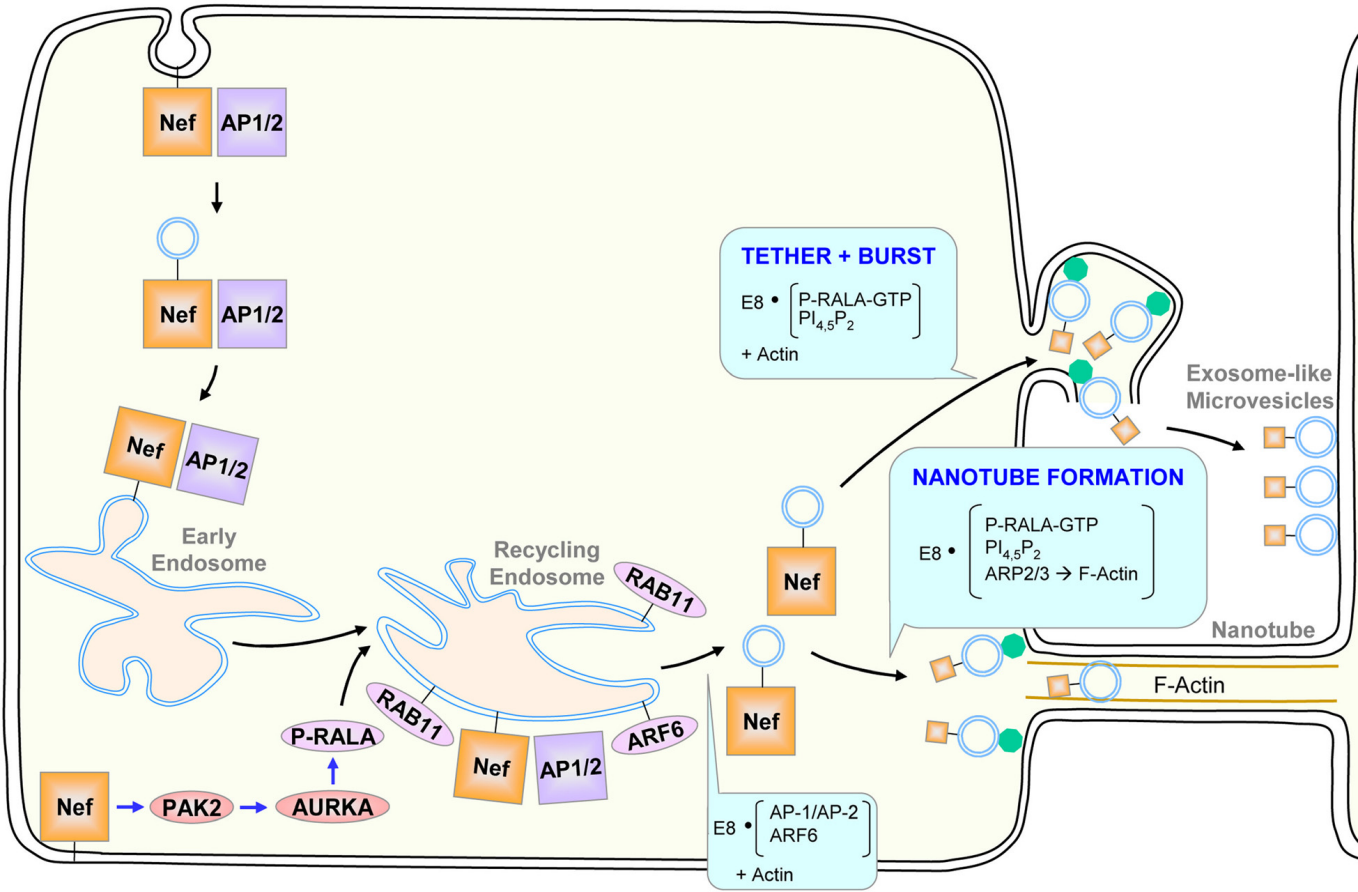
**Proposed model of Nef-mediated enhancement of nanotube formation via the exocyst complex.** Based on proteomics data and protein interaction networks shown in Table 1 and Figure 4, a model of Nef-mediated enhancement of nanotube formation and microvesicle release is shown (described in the text). Briefly, Nef i) traffics to recycling endosomes via enhanced endocytosis, ii) upregulates exocyst complex assembly via Pak2-stimulated Aurora kinase activation (AURKA) [71] and phosphorylation and relocalization of RalA [73], and iii) budding of Nef-bearing vesicles from recycling endosomes [75,76] and enhances iv) Nef secretion in microvesicles (see “tether and burst”) [25] and v) nanotube formation via exocyst complex function, thereby increasing intercellular transfer of Nef. Protein complexes denoted in boxes act at the indicated steps. The exocyst complex is abbreviated as “E8” and depicted as green octagons.

In light of our finding that Nef associates with exocyst complex subunits, protein interaction network modelling (Figure 4), EXOC2 shRNA-mediated inhibition of nanotube formation in Nef-expressing cells, and review of literature, including prior reports that Nef enhances microvesicle secretion and nanotube formation [13,23-25], we propose a potential model to unify these pathways (Figure 6). Our proposed model is based on established Nef-mediated enhancement of endocytosis and exocytosis, and predicted Nef-mediated enhancement of exocyst complex assembly. Nef binding to adapter protein 2 μ-subunit (AP2M1) enhances endocytosis and trafficking of Nef to recycling endosomes [70]. Upon Nef-mediated activation of Pak2, Pak2 activates Aurora-A [71], which phosphorylates a major exocyst complex assembly regulator, RalA GTPase [72,73]. RalA mediates assembly of exocyst complex components, enabling polarized docking with membrane-associated EXOC1 and EXOC7 [58,59,74] on endosomes and/or recycling endosome-derived vesicles. Interaction of Nef with a protein complex that includes the exocyst complex and RalA may therefore lead to Nef-mediated formation of nanotubes. Non-filamentous assembly of actin may lead to formation of nodules filled with exocyst-tethered vesicles, which transfer to bystander cells following plasma membrane rupture. Thus, our findings, together with previous studies [13,25,66], suggest potential involvement of RalA and the exocyst complex in Nef-mediated formation of nanotubes and enhanced release of exosome-like vesicle clusters.

Interactor(s) that mediate Nef association with exocyst complex subunits are not yet clear. One possibility is that Nef may interact directly with a component(s) of the exocyst complex. In this scenario, Nef might function as a viral homolog of a cellular exocyst complex regulator downstream of Pak2 activation. Among the exocyst complex components detected in our mass spectrometry analysis, the greatest number of peptides were derived from EXOC4 (Table 1), the same component identified by Jager *et al.* as a Nef-interacting protein in their proteomic screen [17]. Taken together, these data suggest EXOC4 represents a potential candidate for an exocyst complex protein that may bind directly to Nef. Given predicted protein interaction networks (Figure 4), Nef-AP2M1 binding is another potential means of Nef-exocyst complex association. Co-immunoprecipitation of Nef with Rab11 has been reported [77], raising the alternative possibility that Rab11 might bridge between Nef and the exocyst complex via its interaction with EXOC6 [78]. Our co-immunoprecipitation assays were conducted in lysis buffer containing a concentration of detergent sufficient to disrupt membranes (1% Triton X-100); therefore, it seems unlikely that Nef-exocyst complex association could be attributed to membrane bridging. Both Nef and the exocyst complex are targeted to lipid rafts [30,54,79,80], and both have been linked to nanotube formation [13,66]. As such, Nef and the exocyst complex are likely to co-localize at plasma membrane sites to mediate nanotube formation. Further studies are required to determine whether Nef association with the exocyst complex is direct or indirect, and whether clathrin adapter complex protein(s), Rab11, or other yet unknown protein(s) bridge between Nef and the exocyst complex. RNAi-mediated knockdown experiments targeting nodes of the Nef-exocyst interactome network (Figure 4) will help to elucidate the mechanism(s) by which Nef hijacks exocyst function to enhance nanotube formation. Additionally, future studies utilizing Nef mutants to map determinants of Nef-exocyst association will be important to clarify the functional relationships between Nef-Pak2 and Nef-exocyst interactions.

Several nodes of our Nef-exocyst interactome (Figure 4) are in agreement with results of prior proteomic and RNAi-based studies of HIV interactions with the host cell (Additional file 6: Figure S3). In their proteomic study with Jurkat cells, Jager *et al*. also identified Vav and the clathrin adapter proteins AP1S1A and AP3S1 as Nef-interacting proteins [17]. Furthermore, siRNA screens indicated that AP2M1, PIP5K1C, RAB11A, RalB, and RalBP1 expression is important for HIV-1 replication [81,82]. Therefore, in addition to the role of Nef-mediated enhancement of nanotube formation, it is possible that interaction of HIV-1 proteins with Nef-exocyst interactome components (Figure 4 and Additional file 6: Figure S3) may have broader functional implications for viral replication. Future studies to examine the effects of RNAi-mediated knockdown of EXOC2 and other Nef-exocyst interactome components will be important to understand the contribution of these interactions to HIV replication and pathogenesis.

There are striking similarities between mechanisms by which nanotubes, filopodia, virological synapses, and immunological synapses are formed, which include cytoskeletal polarization, membrane protrusion, and recruitment of proteins to discrete membrane domains that serve as foci for endocytosis and exocytosis [42,66,83-85]. Furthermore, exocyst complex function is required for formation of filopodia and nanotubes [66,85]. Together with our findings that Nef associates with exocyst components and EXOC2 is required for Nef-mediated enhancement of nanotube formation, these observations provide further support for a model in which formation of Nef-induced nanotubes [13] involves Nef-mediated regulation of the exocyst complex.

The relationship of our findings to Nef-mediated enhancement of viral replication and cellular activation remains an open question. Cell-to-cell transfer is an important mode of HIV-1 dissemination within the host; for CD4+ T-cells, intercellular transfer is 2 to 3 logs more efficient at supporting viral replication than HIV-1 infection with cell-free inoculum [42,50]. Nef enhances formation of the virological synapse, which is structurally similar to intercellular nanotubes [42]. Thus, Nef-mediated regulation of the exocyst complex may also play a role in intercellular transmission of HIV-1 via the virological synapse. Exosomes or microvesicles that contain Nef [24] or proinflammatory molecules such as cytokines [86] contribute to cell activation [25] and activation-induced apoptosis [24]. As such, Nef may increase chronic immune activation in part by upregulating exocyst complex function and microvesicle secretion.

## Conclusions

Identification of EXOC1, EXOC2, EXOC3, EXOC4, and EXOC6 as Nef-interacting host cell factors and our finding that EXOC2 depletion abrogates Nef-mediated enhancement of nanotube formation provide a new paradigm for future investigations into Nef-mediated mechanisms involved in nanotube formation, intercellular transfer of Nef, cell-to-cell spread of HIV-1, and acceleration of disease progression. Moreover, this paradigm of exocyst involvement in polarized targeting for intercellular transfer of viral proteins and virions is likely to be relevant for other pathogens as well. For example, HTLV and prions spread from cell to cell via virological synapses and nanotubes, respectively [87,88]. Understanding interactions between viral proteins and exocyst complex components may therefore lead to the discovery of novel strategies to inhibit intercellular spread of diverse pathogens.

## Availability of supporting data

Supporting data are included within the article and its additional files.

## Methods

### Cell culture

Jurkat clone E6-1 cells (ATCC) were maintained in high-glucose RPMI 1640 medium with 0.3 mg/L L-Glutamine (GIBCO), supplemented with 10% fetal bovine serum, 1 mM sodium pyruvate, 14 mM glucose, 55 nM β-mercaptoethanol, and penicillin-streptomycin. HEK293T cells were maintained in DMEM (GIBCO) supplemented with 10% fetal bovine serum and penicillin-streptomycin.

### Lentiviral vector construction and production

pHAGE viral vector stocks were made as follows: Nef genes were inserted into pHAGE EF1α-IRES-zsGreen, kindly provided by the Harvard Gene Therapy Initiative [89], via PCR amplification from pCR3.1 constructs using the primers 5′AAAAGCGGCCGCCACCATGGGTGGCAAGTGGTCAAAA3′ and 5′AAGGATCCTCATGAAGCGTAATCTGGCAC 3′, which add a NotI site and a Kozak sequence to the 5′ end of Nef and a BamHI site to the 3′ end of Nef. GenJet (Signagen) lipid transfection was used to introduce 3 μg of pHAGE vector, 3 μg of the packaging construct pDR89.1 [90], and 0.5 μg of pVSV-G per 5.5 × 10^6^ 293T cells plated 24 h prior in a 10-cm tissue culture plate. Transfection mixtures were removed and replaced with fresh media at 16 h post-transfection. Two fractions of vector-containing supernatants were harvested from each plate: after an additional 24 h, supernatants were removed, stored at +4°C, replaced with fresh media, and then pooled with supernatants collected 24 h hence to generate viral stocks. pHAGE vector virion stocks were assayed for RT activity as described previously [91]. pLKO.1 vector virions were made via the same process, substituting pLKO.1-shRNA-puro^R^ constructs (Sigma, Mission shRNA) in lieu of pHAGE.

### Lentiviral vector transduction of Jurkat cells

Twenty million (for immunoprecipitation) or 8 × 10^6^ (for nanotube formation assay) Jurkat E6-1 cells were pHAGE-transduced with cpm/cell units of RT activity normalized to yield populations of matched ZsGreen fluorescence (~0.2 cpm/cell and 0.05 cpm/cell for pHAGE-Nef-IRES-zsGreen and pHAGE-IRES-zsGreen vector virions, respectively). At 4 h post-transduction, cells were washed twice with PBS and incubated in fresh media overnight (for nanotube formation assay), or for an additional 44 h (for immunoprecipitation and mass spectrometry).

### Nef immunoprecipitation from Jurkat cell lysates

At 48 h post-transduction, Jurkat E6-1 cells transduced with pHAGE vector virions as described above were lysed in 1 mL of ice-cold lysis buffer with protease and phosphatase inhibitors [CLB + PPI: 50 mM Tris HCl pH 7.5, 150 mM NaCl, 1% Triton, 1 mM EDTA, 1X Complete EDTA-free protease inhibitor cocktail (Roche), 1X PhosSTOP phosphatase inhibitor cocktail (Roche)] and 0.45 μm spin-filtered (Millipore UFC40HV00). Immunocomplexes were captured with α-HA-agarose (Santa Cruz, clone F-7) during overnight incubation with rotation at 4°C, rinsed 5 times with CLB + PPI and 3 times with 1X PBS, and eluted in PBS with 250 μg/mL HA peptide (Sigma, #I2149) and 1X Complete EDTA-free protease inhibitor cocktail (Roche) at room temperature.

### Mass spectrometry

Jurkat E6-1 cells (20 × 10^6^) were transduced with pHAGE vector virions and lysed for immunoprecipitation with α-HA-conjugated agarose as described above.

Nef-HA immunocomplexes were eluted with HA peptide, trichloroacetic acid-precipitated, trypsinized, purified, and analyzed via LC-MS/MS on a Velos LTQ Linear ion trap mass spectrometer at the Harvard Medical School Taplin Biological Mass Spectrometry Facility.

### Gel electrophoresis and silver staining

Prior to co-immunoprecipitation for mass spectrometry, 1% of the input was resolved via 4–12% Bis-Tris SDS-PAGE in MOPS buffer (Novex) and silverstained according to the manufacturer’s protocol (SilverQuest, Invitrogen/Life Technologies).

### Immunoblotting

Samples were subjected to SDS-PAGE (4–12% Bis-Tris in 1X MOPS, Novex) and transferred to PVDF (0.2 μm, Bio-rad) for immunoblotting. Blots were blocked with protein-free T20 blocking buffer (Thermo Scientific), and then probed with α-HA-HRP (1:500, Roche), mouse α-β-tubulin (1:2000, Sigma-Aldrich), rabbit α-PAK2 (1:1000, Cell Signaling), rabbit α-EXOC1 (1:250, Proteintech Group), rabbit α-EXOC2 (1:250, Proteintech Group), mouse α-EXOC3 (1:250, Enzo Life Sciences, clone 9H5), or mouse α-EXOC4 (1:1000, Enzo Life Sciences, clone 14G1), and α-mouse or α-rabbit IgG (both Santa Cruz, 1:5000) in protein-free T20 blocking buffer. Immunoblots were washed and visualized via enhanced chemiluminescence (Thermo Fisher) and autoradiography (Kodak, XAR film). Signal intensity for EXOC2 was quantitated using ImageJ software (NIH, v1.45p), and normalized to the β-tubulin loading control.

### FACS analysis

Jurkat E6-1 cells (4 × 10^5^) were washed twice with cold PBS containing 2 mM EDTA (PBS-E) and resuspended in 200 μl of PBS-E containing 2% FBS and 5 μl of CD25-PE (BD Pharmingen). Cells and antibody were incubated at 4°C for 45 min. Cells were washed twice with PBS-E, resuspended in PBS and analyzed on a BD FACSCanto II flow cytometer. FACS data was analyzed using BD FACSDiva software.

### Network analysis of the Nef-exocyst interactome

Exocyst complex proteins identified via mass spectrometry analysis of Nef immunocomplexes, known regulators of exocyst complex function, and known Nef-interacting proteins were input into Ingenuity Pathway Analysis and STRING to generate network diagrams of the Nef-exocyst interactome. The IPA bioinformatic analysis suite maps the identifier for each input molecule to its corresponding object in Ingenuity’s Knowledge Base, overlays network eligible molecules onto a global molecular network developed from information contained in Ingenuity’s Knowledge Base, and then algorithmically generates networks of network eligible molecules based on their connectivity http://www.ingenuity.com. STRING assigns confidence scores using presence of proteins in the same Kyoto Encyclopedia of Genes and Genomes (KEGG) pathway as a benchmark to judge authenticity of a given association, and computes combined confidence scores as the joint probability of the probabilities from the different evidence channels, correcting for the probability of randomly observing an interaction [64].

### Generation of shRNA knockdown cells

Jurkat E6-1 cells were transduced with VSV-G pseudotyped pLKO.1-shRNA-UbiC-*puro*^*R*^ vector virions as described above, and cells expressing shRNAs were selected with 1 ug/mL puromycin for 8 days beginning at 48 h post-transduction. Puromycin selection was removed for 5 days prior to fixation of nanotubes. pLKO.1 constructs encoding shRNAs against human EXOC2 (TRCN0000289958, CCGGCGTGGCACATATTGAAGCATTCTCGAGAATGCTTCAATATGTGCCACGTTTTTG) or a non-human control transcript (SHC-002) were obtained from Sigma (Mission shRNA).

### Nanotube formation assay

Jurkat shRNA knockdown cells were generated and pHAGE-transduced as described above, stimulated with 1 μg/ml PHA-P for 1 h at 24 h post-pHAGE transduction, washed twice with cold PBS, and resuspended at 2 x 10^6^ cells/mL in pre-warmed 50% conditioned media. 22 mm x 22 mm coverslips (Fisher) were ethanol-sterilized, coated with 10 ug/mL fibronectin (Sigma, #F4759), dried overnight and washed 5 times with room temperature PBS and once with media immediately prior to plating 250 ul of cell suspension per coverslip. After 5 h incubation at 37°C, cells were fixed and stained as follows: 0.1% glutaraldehyde/2% formaldehyde (EM-grade, Sigma and Thermo Scientific) in PBS for 1 min at RT, Cytofix/Cytoperm (BD Bioscience) for 5 min at 37°C, 2 × 5 min incubations in 50 mM NH_4_Cl/20 mM glycine in PBS for 5 min at 37°C, 2 × 1X PBS washes, 20 min at RT with 200 ul of 1:40 Alexa Fluor 555-conjugated phalloidin (Molecular Probes) in PBS, and 2 × 1X PBS washes. Coverslips were mounted with ProLong Gold Mounting Medium (Molecular Probes), and cured for 24 h at RT prior to imaging. An aliquot of ~ 4 × 10^6^ reserved, unstimulated cells was washed twice with cold PBS at 32 h post-pHAGE transduction and ~5% of these cells were FACS analyzed for ZsGreen expression, while the remainder were lysed for western blotting and quantitation of EXOC2 knockdown.

### Confocal fluorescence microsopy

Slides of fixed cells prepared in triplicate as described above were imaged using the 40X objective of a Zeiss LSM 510 META scanning confocal microscope and Zeiss LSM imaging software at the Harvard NeuroDiscovery Center Optical Imaging Core Facility. ZsGreen-positive cells were counted by using an ImageJ (NIH, v1.45p) macro to analyze approximately 30 fields per sample at 40X magnification. The numbers of ZsGreen-positive cells counted per sample were: 1441 cells for shEXOC2-Nef, 2048 for shEXOC2 + Nef, 2997 for shControl-Nef, and 1409 for shControl + Nef. The percentage of Nef-expressing cells that formed nanotubes and nanotube-like structures was calculated by manually counting the number of connected cells and dividing the result by the total number of ZsGreen-positive cells for a given sample. Percentages are reported as mean ± SEM.

### Statistical analysis

Differences between pairs of groups were analyzed by the Mann–Whitney *U* test. Differences were considered significant at p ≤ 0.05.

## Abbreviations

AP2M1, μ-subunit of clathrin adapter protein 2 complex; ARHGEF7, β-PIX; AURKA, Aurora-A kinase; β-PIX, Pak-interacting exchange factor β; CLB + PPI, ice-cold lysis buffer with protease and phosphatase inhibitors; CompPASS, Comparative Proteomic Analysis Software Suite; EXOC, exocyst complex component; IPA, Ingenuity Pathway Analysis; LC-MS/MS, liquid chromatography-coupled tandem mass spectrometry; MiST, Mass Spectrometry interaction Statistics; MVB, multivesicular body; Pak2, p21-activated kinase 2; PHA, phytohemagglutinin; PIP5K1C, phosphatidylinositol-4-phosphate 5-kinase type I γ; PMA, phorbol myristate acetate; RT, reverse transcriptase; SFKs, Src-family kinases; shRNA, short haipin RNA; STRING, Search Tool for the Retrieval of Interacting Genes/Proteins; TCR, T-cell receptor.

## Competing interests

The authors declare that they have no competing interests.

## Authors’ contributions

JM, KCO, KAA, and DG designed research; JM and KCO performed research; KAA generated 5C-7 Nef and conducted preliminary experiments; JM, DG, and VM analyzed data; and JM and DG wrote the paper. All authors read and approved the final manuscript.

## Supplementary Material

Additional file 1: Table S1. **Proteins detected via LC-MS/MS in negative control, wild-type Nef, and mutant Nef samples.** All proteins detected via LC-MS/MS analysis of trichloroacetic acid-precipitated HA immunocomplexes from FACS-sorted or unsorted Jurkat cells transduced to express the denoted HA-tagged Nef or empty pHAGE vector are shown alphabetically for side-by-side comparison. “Number of peptides” indicates the number of distinct peptides from a single protein detected. International Protein Index identification codes are provided in the “IPI” column. Complete alphabetical listing of proteins detected via mass spectrometry analysis for side-by-side comparision. Click here for file

Additional file 2: Table S2. **CompPASS statistical analysis of proteins detected via LC-MS/MS in negative control, wild-type Nef, and mutant Nef samples.** All proteins detected via LC-MS/MS analysis of trichloroacetic acid-precipitated HA immunocomplexes from FACS-sorted or unsorted Jurkat cells transduced to express the denoted HA-tagged Nef or empty pHAGE vector are shown, grouped sequentially by descending D^N^-score (defined in the text and in [55]). “TSC” column lists the number of distinct peptides detected from a single protein. “Ratio” denotes the proportion of mass spectrometry datasets in the CompPASS database in which a given interactor was detected. Complete listing of proteins detected via mass spectrometry analysis with CompPASS statistics, listed sequentially by descending D^N^-score. Click here for file

Additional file 3: Figure S1. **Statistical significance of protein associations detected via LC-MS/MS analysis of wild-type 5C Nef samples.** Plotting D^N^- versus Z-scores, as in [55], indicates that Nef-association of select proteins detected exclusively in wild-type Nef samples (red data points) is significant based on Z-score and/or D^N^-score. In a previous study conducted by the creators of CompPASS, Z ≥ 4 and D^N^ ≥ 1 were used as significance thresholds [55]. In instances where D^N^ is slightly less than 1, overwhelmingly high Z-scores compensate for the D^N^-score and indicate that statistical significance is likely. Plot of D^N^ versus Z-scores for proteins detected via LC-MS/MS analysis of wild-type 5C-Nef samples. Click here for file

Additional file 4: Table S3 **Protein-protein relationship scores for associations in STRING network.** Scores assigned to protein-protein association relationships in the STRING knowledgebase are shown for proteins input into STRING to generate Figure 4B (described in the text). Confidence scores for protein-protein associations in Figure 4B STRING network. Click here for file

Additional file 5: Figure S2 **Confocal image gallery of nanotubes, nanotube-like structures, and filopodia in Jurkat cells expressing empty pHAGE or pHAGE-Nef.** Jurkat E6-1 cells transduced with vector virions bearing pLKO.1- control shRNA followed by pHAGE-Nef-IRES-ZsGreen or empty pHAGE, as described in Figure 5, were fixed and stained with Alexa Fluor 555-conjugated phalloidin. Nanotubes, nanotube-like structures, and filopodia were visualized via confocal microscopy; selected zoomed images of these structures are shown. Scale bars, 5 μm. Gallery of additional confocal images of pHAGE and pHAGE-Nef-expressing Jurkat cells showing various phenotypes discussed in Figure 5. Click here for file

Additional file 6: Figure S3 **Comparison of proteins identified in Nef proteomic and HIV RNAi screens.** Datasets from Nef mass spectrometry analyses conducted by Mukerji et al. in the present study and Jager *et al*. (Krogan Lab) [17], together with results of RNAi screens performed by Brass *et al*., Konig *et al*., Zhou *et al*., and Yeung *et al*. [81,82,92,93] were input into Venny [94] to generate a Venn diagram comparison. The Mukerji Nef dataset consists of 10 proteins identified exclusively in 5C Nef immunocomplexes (Table 1). A minimum MiST score threshold of 7.07 × 10^-9^ was applied to the Jager *et al*. dataset of Nef-interacting proteins in Jurkat cells (see Supplementary Data 3 in [17]). Venn diagram comparison of Nef-interacting proteins identified by mass spectrometry analysis of Nef immunocomplexes in the present study and Jager *et al*., and molecules identified as important for HIV replication in RNAi screens by Brass *et al*., Konig *et al*., Zhou *et al*., and Yeung *et al*. Click here for file
