# Supplementary figures and images for: Proteomic analysis of HIV-1 Nef cellular binding partners reveals a role for exocyst complex proteins in mediating enhancement of intercellular nanotube formation
Source: Retrovirology. 2012 Jun 22;9:33. doi: 10.1186/1742-4690-9-33 (PMC3382630; doi:10.1186/1742-4690-9-33)

## Slide 1
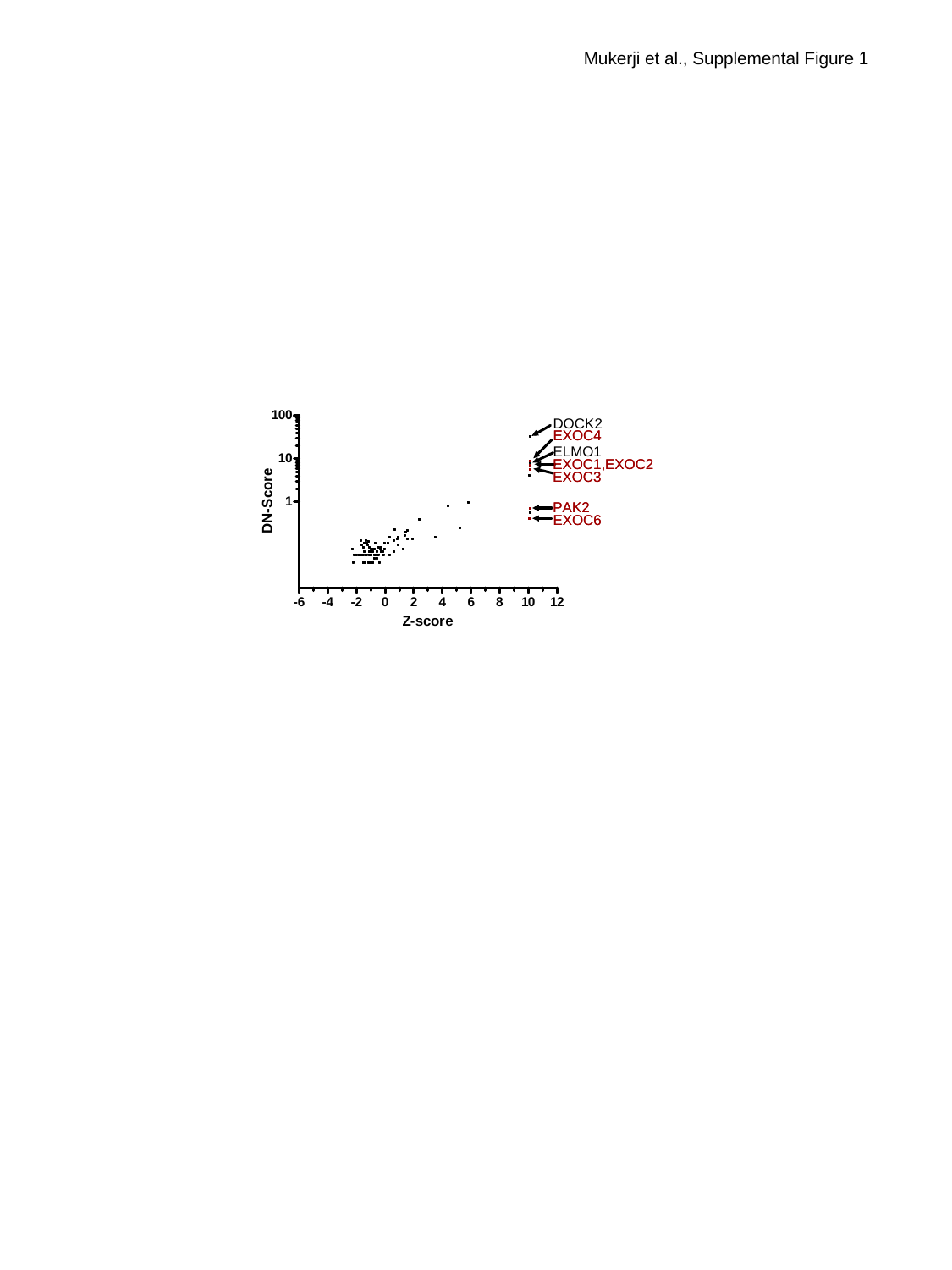

Mukerji et al., Supplemental Figure 1

Supplement: Additional file 3: Figure S1. — Statistical significance of protein associations detected via LC-MS/MS analysis of wild-type 5C Nef samples. Plotting DN- versus Z-scores, as in [55], indicates that Nef-association of select proteins detected exclusively in wild-type Nef samples (red data points) is significant based on Z-score and/or DN-score. In a previous study conducted by the creators of CompPASS, Z ≥ 4 and DN ≥ 1 were used as significance thresholds [55]. In instances where DN is slightly less than 1, overwhelmingly high Z-scores compensate for the DN-score and indicate that statistical significance is likely. Plot of DN versus Z-scores for proteins detected via LC-MS/MS analysis of wild-type 5C-Nef samples. [file 1742-4690-9-33-S3.ppt]
