# Supplementary material for: Proteomic analysis of HIV-1 Nef cellular binding partners reveals a role for exocyst complex proteins in mediating enhancement of intercellular nanotube formation
Source: Retrovirology. 2012 Jun 22;9:33. doi: 10.1186/1742-4690-9-33 (PMC3382630; doi:10.1186/1742-4690-9-33)
Supplement: Additional file 5: Figure S2 — Confocal image gallery of nanotubes, nanotube-like structures, and filopodia in Jurkat cells expressing empty pHAGE or pHAGE-Nef. Jurkat E6-1 cells transduced with vector virions bearing pLKO.1- control shRNA followed by pHAGE-Nef-IRES-ZsGreen or empty pHAGE, as described in Figure 5, were fixed and stained with Alexa Fluor 555-conjugated phalloidin. Nanotubes, nanotube-like structures, and filopodia were visualized via confocal microscopy; selected zoomed images of these structures are shown. Scale bars, 5 μm. Gallery of additional confocal images of pHAGE and pHAGE-Nef-expressing Jurkat cells showing various phenotypes discussed in Figure 5. [file 1742-4690-9-33-S5.ppt]

## Slide 1
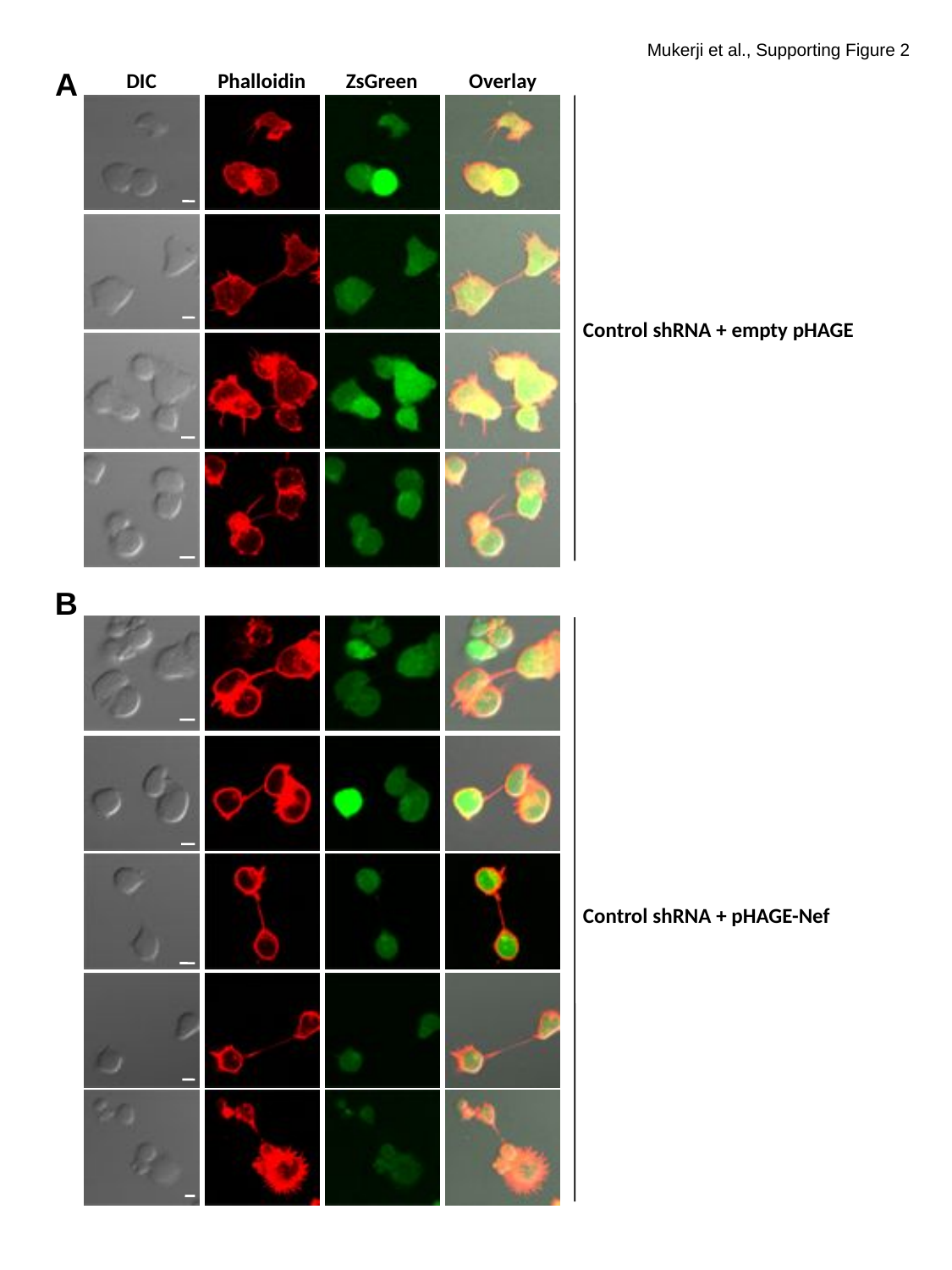

Mukerji et al., Supporting Figure 2
A
DIC
Phalloidin
ZsGreen
Overlay
Control shRNA + empty pHAGE
B
Control shRNA + pHAGE-Nef
