# Supplementary material for: Proteomic analysis of HIV-1 Nef cellular binding partners reveals a role for exocyst complex proteins in mediating enhancement of intercellular nanotube formation
Source: Retrovirology. 2012 Jun 22;9:33. doi: 10.1186/1742-4690-9-33 (PMC3382630; doi:10.1186/1742-4690-9-33)
Supplement: Additional file 6: Figure S3 — Comparison of proteins identified in Nef proteomic and HIV RNAi screens. Datasets from Nef mass spectrometry analyses conducted by Mukerji et al. in the present study and Jager et al. (Krogan Lab) [17], together with results of RNAi screens performed by Brass et al., Konig et al., Zhou et al., and Yeung et al. [81,82,92,93] were input into Venny [94] to generate a Venn diagram comparison. The Mukerji Nef dataset consists of 10 proteins identified exclusively in 5C Nef immunocomplexes (Table 1). A minimum MiST score threshold of 7.07 × 10^-9^ was applied to the Jager et al. dataset of Nef-interacting proteins in Jurkat cells (see Supplementary Data 3 in [17]). Venn diagram comparison of Nef-interacting proteins identified by mass spectrometry analysis of Nef immunocomplexes in the present study and Jager et al., and molecules identified as important for HIV replication in RNAi screens by Brass et al., Konig et al., Zhou et al., and Yeung et al. [file 1742-4690-9-33-S6.ppt]

## Slide 1
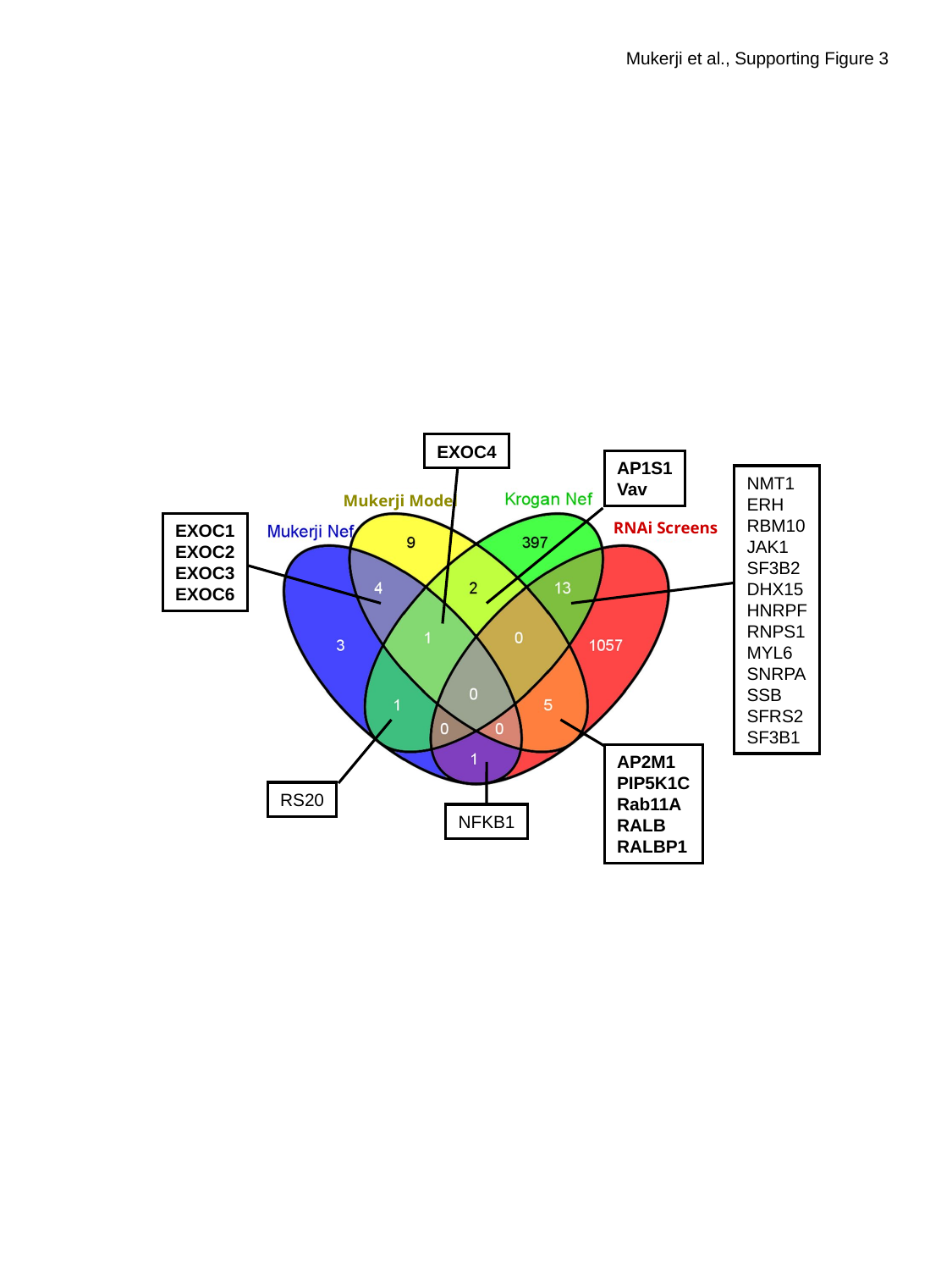

Mukerji et al., Supporting Figure 3
EXOC4
AP1S1
Vav
NMT1
ERH
RBM10
JAK1
SF3B2
DHX15
HNRPF
RNPS1
MYL6
SNRPA
SSB
SFRS2
SF3B1
Mukerji Model
RNAi Screens
EXOC1
EXOC2
EXOC3
EXOC6
AP2M1
PIP5K1C
Rab11A
RALB
RALBP1
RS20
NFKB1
